# Racecadotril in the treatment of acute diarrhea in children: a systematic, comprehensive review and meta-analysis of randomized controlled trials

**DOI:** 10.1186/s12887-018-1095-x

**Published:** 2018-04-03

**Authors:** Marion Eberlin, Min Chen, Tobias Mueck, Jan Däbritz

**Affiliations:** 1 Department of Medical Affairs CHC GSA, Sanofi-Aventis Deutschland GmbH, Frankfurt am Main, Germany; 2 Department of Anesthesiology, Wuhan Union Hospital, Wuhan, China; 3 Department of Pediatrics, University Hospital Rostock, Rostock, Germany; 4 Center for Immunobiology, Blizard Institute, Barts Cancer Institute, The Barts and the London School of Medicine & Dentistry, Queen Mary University, London, UK

**Keywords:** Diarrhea, Children, Racecadotril, Loperamide, Meta-analysis, Probiotic, Smectite

## Abstract

**Background:**

Racecadotril is a guideline-recommended option for the treatment of acute diarrhea in children but existing guidelines and previous reviews of the field are based on a small fraction of published evidence. Therefore, we have performed a systematic search for randomized controlled trials evaluating racecadotril as add-on or in comparison to other treatments.

**Methods:**

A search was performed in PubMed, Scopus and Google Scholar without limits about country of origin or reporting language. A meta-analysis was conducted for the five most frequently used efficacy parameters.

**Results:**

We have retrieved 58 trials, from nine countries including six in comparison to placebo, 15 in comparison to various active treatments and 41 as add-on to various standard treatments (some multi-armed studies allowing more than one comparison). Trials used 45 distinct efficacy parameters, most often time to cure, % of cured children after 3 days of treatment, global efficacy and number of stools on second day of treatment. Racecadotril was superior to comparator treatments in outpatients and hospitalized patients with a high degree of consistency as confirmed by meta-analysis for the five most frequently used outcome parameters. For instance, it reduced time to cure from 106.2 h to 78.2 h (mean reduction 28.0 h; *P* < 0.0001 in 24 studies reporting on this parameter). Tolerability of racecadotril was comparable to that of placebo (10.4% vs. 10.6% adverse events incidence) or that of active comparator treatments other than loperamide (2.4% in both groups).

**Conclusions:**

Based on a comprehensive review of the existing evidence, we conclude that racecadotril is more efficacious than other treatments except for loperamide and has a tolerability similar to placebo and better than loperamide. These findings support the use of racecadotril in the treatment of acute diarrhea in children.

## Background

Acute diarrhea in children is a global health problem with an estimated 2 billion episodes each year; an estimated 1.9 million children die from the condition, mostly in developing countries, amounting to 18% of all deaths in children under the age of 5 years [1]. Seventy eight percent of these fatalities occur in Africa and Southeast Asia. In developed countries, acute diarrhea is usually but not always a mild disease only rarely associated with mortality but with a substantial number of hospitalizations and high costs [2]. Oral rehydration treatment (ORT) is the cornerstone of treatment of acute diarrhea and its widespread adoption has improved prognosis of the condition over the past 30 years [1]. Several medications are available that reduce symptom severity and/or shorten duration of a diarrheic episode, including zinc, adsorptive agents such as charcoal and smectite, probiotics, anti-bacterial and anti-viral drugs, and the opioid receptor agonist loperamide [2], although use of the latter is contra-indicated in infants younger than 24 months [3] and no longer recommended in recent guidelines [2].

Racecadotril is a more recent addition to the armamentarium for the treatment of acute diarrhea in children [4–6]. We have comprehensively reviewed the pharmacodynamics and pharmacokinetics of racecadotril and its metabolites elsewhere [7]. In short, racecadotril is an inhibitor of the endorphin-metabolizing enzyme neutral endopeptidase (NEP; EC 3.4.24.11) that is also known under the name enkephalinase. Racecadotril is rapidly metabolized to thiorphan [8]. Its stereoisomers S-thiorphan, also known as ecadotril or sinorphan, and R-thiorphan, also known as retorphan or dexecadotril, are both considerably more potent NEP inhibitors than racecadotril or acetyl-thiorphan, which is an alternative metabolite of racecadotril [9]. Unless specifically indicated otherwise, ‘racecadotril’ collectively refers to the parent compound and its active metabolites in the rest of this article. NEP inhibition by racecadotril and its metabolites increases levels of endogenous enkephalines, which potently inhibits secretion in the gut with only little effect on motility [10]. Racecadotril has been shown to inhibit rotavirus-induced secretion in Caco-2 cells [11] and cholera toxin-induced secretion in canine [12] and human jejunum [13] but has only little effect on basal secretion. Racecadotril did not alter gastrointestinal transit times in rat or mice [14] or healthy human volunteers [15, 16], which is in contrast to the effects of the opioid receptor agonist loperamide. Based on its anti-secretory activity against pathological agents, racecadotril has been shown to mitigate castor oil-induced diarrhea in rats [14] and healthy human volunteers [17]. Accordingly, racecadotril did not alter *E. coli* content in proximal jejunum and reduced it in stool of newborn piglets, whereas the gastrointestinal motility inhibitor loperamide increased the *E. coli* content in jejunum and reduced it in stool [18]. Taken together, these pharmacological properties should make racecadotril an effective agent for the treatment of acute diarrhea with little potential for retention of infectious agent or rebound constipation.

**Fig. 1:**
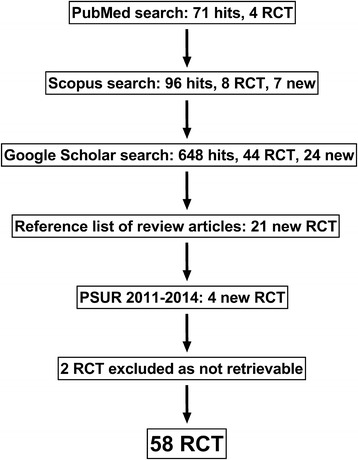
Flow chart of retrieved studies. For each source, we show number of hits and randomized controlled trials (RCTs) as well as number of RCT not retrieved by preceding searches (“new”). PSUR, periodic safety update report

The efficacy and safety of racecadotril in the treatment of acute diarrhea in children has been the subject of several reviews and meta-analysis [19–24]. Based on such data, international guidelines recommend racecadotril as a treatment option in children with acute diarrhea [1, 2, 25]. However, previous reviews of the field had language and/or cultural limitations and only focused on only a small fraction of the existing literature (2–9 studies largely excluding those from China or 19 studies only from China). In a recent systematic search for studies of racecadotril in the treatment of acute diarrhea in children with no limitation for language of the report, we have identified 57 randomized trials, i.e. more than three times as many as the most comprehensive previously published review of the field (Fig. 1). Therefore, we have performed a systematic review of reported randomized trials on the effects of racecadotril in children with acute diarrhea and performed a meta-analysis of the five most frequently used efficacy parameters. To the best of our knowledge, this is the first truly comprehensive summary of such studies that did not limit inclusion based on country where a study was performed or language in which it was reported. The effects of racecadotril in comparison to other treatments of acute diarrhea in adults have been comprehensively reviewed elsewhere [26].

## Methods

The present analysis follows the PRISMA guidelines for systematic reviews (www.prisma-statement.org). It is based on dedicated literature searches completed in September 2016 in PubMed, Scopus and Google Scholar for the key word combination ‘racecadotril’ and ‘diarrhea’/‘diarhoea’ (Fig. 1). We included original studies reporting randomized clinical trials evaluating racecadotril in children with acute diarrhea, either as addition to standard treatment or in comparison to an active treatment. To this end, we originally defined children as participants under the age of 18 years, but it turned out that all retrieved studies had limited inclusion to an age of up to only 10 years, except for one trial 6 years or less (Table 1). Reference lists of retrieved original articles as well as review articles were screened for additional publications. Studies reported as abstracts were also included, if a dedicated search could not identify a corresponding full paper. Screening of identified hits was primarily done at the level of article title; if that was ambiguous, the abstract was screened; if that still remained ambiguous the full text was analyzed. There were few studies in which one treatment arm was a racecadotril + X combination, whereas the other treatment arm was Y. These were excluded because they do not allow direct conclusions on the efficacy and safety of racecadotril. In contrast, studies in which one treatment arm was a racecadotril + X combination and the other treatment arm was X alone were included and are referred to as “add-on studies” in our manuscript. Non-randomized studies have not been included in any of the analyses presented in tables and figures. However, we sometimes used them to put RCT findings into perspective; for instance, an observational study in Venezuela documenting outcomes of treatment with racecadotril in 3873 children [27].

**Table 1:** Randomized studies included in analysis

Background	Comparator	Country	Age, months	N per arm	Comment	Reference
Blinded placebo-controlled studies						
ORT	placebo	Peru	3–35	67–68	DB, HB, boys only	[40]
ORT	placebo	France	3–48	82–84	DB, HB	[38]
ORT	placebo	India	< 60	30	DB, HB, ABS	[42]
ORT or IRT	placebo	China	< 24	39	DB, HB	[44]
ORT and/or IRT, zinc	placebo	Kenya	3–60	57–58	DB, HB	[39]
ORT	placebo	Ecuador	3–36	34–45	SB, HB	[35]
Open-label add-on studies						
ORT or IRT	–	France	3–36	81–83	HB	[36]
ORT or IRT	–	China	4–36	24–36	HB	[45]
ORT	–	Spain	3–36	70–78	HB	[33]
ORT	–	China	3–36	24	Setting n.r.	[46]
ORT	–	Spain	3–36	94	HB	[34]
ORT	–	Egypt	12–72	30	HB	[41] study I
ORT	–	Egypt	12–72	15	OB	[41] study IIc
ORT, smectite	–	China	1–30	30–35	HB	[47]
ORT, smectite	–	China	6–11	58	OB	[48]
ORT or IRT, smectite	–	China	2–36	89	HB, 3-armed	[30]
ORT, smectite	–	China	3–36	124–134	HB, OB	[49]
ORT, smectite	–	China	3–36	50–60	Setting n.r.	[50]
ORT, smectite, PB	–	China	2–36	43–53	HB, OB, 3-armed	[29]
ORT, smectite, PB	–	China	4–27	113–165	Setting n.r.	[51]
ORT, smectite, PB	–	China	3–36	60	Setting n.r.	[53]
ORT, smectite, PB	–	China	6–24	59–62	Setting n.r.	[54]
ORT, smectite, PB	–	China	3–36	30–34	Setting n.r.	[55]
ORT, smectite, PB	–	China	< 24	50	Setting n.r.	[56]
ORT, smectite, PB	–	China	5–27	22–43	Setting n.r.	[52]
ORT, smectite, PB	–	China	2–36	57–68	HB	[57]
ORT, smectite, PB	–	China	2–24	50	Setting n.r.	[58]
ORT or IRT, smectite, PB	–	China	5–24	53	HB	[59]
ORT, smectite, PB	–	China	2–36	56	OB and HB	[60]
ORT or IRT, smectite, PB	–	China	4–24	40	HB	[61]
ORT, smectite, PB	–	China	1–24	43–45	HB	[62]
ORT, smectite, PB		China	3–24	52–56	HB	[63]
ORT, smectite, PB	–	China	6–24	28–30	OB	[64]
IRT, AV	–	China	6–36	48–54	HB	[66]
ORT or IRT, AV	–	China	6–48	40	HB, 4-armed	[31]
ORT or IRT, AV	–	China	2–36	66–69	HB	[67]
ORT, smectite, ribaverine	–	China	6–24	50	Setting n.r.	[65]
ORT, smectite, AV	–	China	18*	56	HB	[68]
ORT, PB, AV	–	China	1–24	38–42	Setting n.r.	[69]
ORT, PB, AV	–	China	3–24	52–68	Setting n.r.	[70]
ORT or IRT, smectite, PB, AV	–	China	6–36	36–44	OB and HB	[71]
ORT, AV, AIN	–	China	7–36	69	Setting n.r.	[72]
ORT, nitazoxanide	–	Egypt	12–72	15	OB	[41] study IIa
ORT, metronidazole	–	Egypt	12–72	15	OB	[41] study IIb
ORT, PB, AI	–	China	12–60	43	HB	[73]
ORT, AI	–	China	6–24	50	OB and HB, 4-armed	[32]
Not fully specified	–	China	4–36	60	HB	[74]
Blinded actively controlled studies						
	Loperamide	France	24–120	50–52	DB, setting n.r.	[37]
Open-label actively controller				studies		
ORT, IRT, PB	Smectite	China	6–24	60	HB	[79]
ORT	Smectite	China	1–36	150	HB	[77]
ORT or IRT	smectite	China	2–36	89	HB, 3-armed	[30]
IRT, PB	smectite	China	6–24	42–44	HB	[78]
ORT, PB, (AB)	Smectite	China	2–60	56–58	OB	[76]
ORT	PB	China	4–24	50	Setting n.r.	[81]
ORT or IRT, AV	PB	China	6–48	40	HB, 4-armed	[31]
IRT	PB	China	6–24	40–42	Setting n.r.	[80]
ORT, AI	PB	China	6–24	50	OB and HB, 4-armed	[32]
ORT	Smectite + PB	China	2–24	41–43	Setting n.r.	[83]
ORT	Smectite + PB	China	2–36	43–53	HB, OB, 3-armed	[29]
ORT or IRT, AV	Smectite + PB	China	2–36	56	HB, OB	[82]
ORT	Kaolin/pectin	Guatemala	3–71	25	OB	[84]
ORT, PB, smectite	Lactose-free diet	China	4–36	34–38	OB, 3-armed	[85]

*AB* antibiotic not otherwise specified, *ABS* study reported in abstract form only, *AI* anti-infectious drug not otherwise specified, *AIN* anti-inflammatory drug not otherwise specified, *AV* anti-viral not otherwise specified, *DB* doubleblind, *HB* hospital based, *IRT* intravenous rehydration treatment; *n.r*. not reported, *OB* office-based, *ORT* oral rehydration treatment, *PB* probiotic, *SB* single-blind. *mean value, range not reported. Note that some 3- or 4-armed studies are listed twice, once for add-on and once for active control comparator

Our search has identified a total of 60 randomized studies. However, no information on study design and results could be retrieved for two of these despite intensive efforts; one is a master thesis by Nassar from the University of Cairo (Egypt) and the other a paper by Gutierrez-Castrellon cited as ‘in press’ in a review by this author [28] but never having appeared as the journal apparently has ceased to exist. Therefore, the present analysis is based on a total of 58 distinct studies described in 55 reports (Table 1); this included 4 reports from 3- or 4-armed studies [29–32], allowing comparison of racecadotril treatment to more than one comparator (Table 1). As there were no language limitations of the search, we have retrieved articles published in Chinese (*n* = 44), English (*n* = 10), Spanish (*n* = 3), and French (*n* = 1). Articles published in Chinese, English or French were directly analyzed; those published in Spanish were translated into English by a professional translator or extracted by a colleague fluent in that language. From each report, we extracted the following data (Table 1):

- Country of origin and reporting language
- Background and comparator treatment
- Presence of randomization
- Presence of blinding (double-blind, single-blind, open-label)
- Range and mean age of patients
- Number of patients per study arm
- Treatment setting (hospital-based including emergency room vs. office-based)
- Efficacy parameter (Table 2)
- Tolerability and safety parameters (Table 3)

**Table 2:** Reported efficacy parameters in randomized studies with racecadotril in the treatment of acute diarrhea in children. Note that most studies have reported multiple endpoints but only 12 had a pre-specified primary endpoint. For details see main text

Efficacy parameter	Number of studies total	Number of studies as primary endpoint
Global and qualitative efficacy parameters		
Day 3 cure/improved/no change	11	–
Day 3 markedly effective/effective/ineffective	30	–
Day 5 markedly effective/effective/ineffective	2	–
Stool number, quality and amount		
24 h number of stools	9	–
48 h number of stools	12	4
72 h number of stools	4	–
7–10 day number of stools	2	–
Total number of stools until cure	3	1
24 h stool output (g)	2	–
48 h stool output (g)	3	3
Total stool output (g) until cure	2	–
48 h % of patients with watery stools	2	–
Day 5 negative stool culture	1	–
Day 7 % of patients with solid stool	1	–
Kaplan-Meier analysis of unresolved diarrhea over time	2	–
Measures of duration of disease and fluid replacement		
Total duration of diarrhea (incl. Pre-treatment)	7	–
Time to cure	22	3
Day 1 cure rate	1	–
Day 2 cure rate	3	–
Day 3 cure rate	41	–
Day 5 cure rate	4	–
Day 7 cure rate	4	–
Duration of fever	2	–
Time to correction of dehydration	2	–
Day 2 need for ORT	1	–
Day 3 need for ORT	1	–
Total volume of ORT requirement	4	–
Need for unscheduled IRT	2	–
Duration of treatment	6	–
Doctor visits and social outcomes		
2^nd^ emergency room visit	3	1
Day 2 emergency room visit	1	–
Day 7 emergency room visit	1	–
Number of doctor visits during follow-up	1	–
Duration of hospitalization	4	–
% hospitalized after 24 h	1	–
% hospitalized after 48 h	1	–
Need for secondary hospitalization	1	–
Nursery/school attendance	1	–
Degree of patient satisfaction	1	–
Other efficacy parameters		
% resolution of vomiting	1	–
Need for additional medication	1	–
Body weight at end of treatment	1	–
Na^+^/K^+^ ratio in urine	1	–
IL-1, IL-8 and IL-12 in serum	1	–
Recurrence rate	1	–

**Table 3:** Adverse event (AE) incidence in randomized studies with racecadotril as compared to various comparators. Data are based on 41 studies from Table 1 that have provided treatment-specific AE data. AE incidence in a large observational study is shown for comparison [27]. For details see section 5

	Comparator			Racecadotril		
	# of patients	# of AE	% of AE	# of patients	# of AE	% of AE
All randomized studies	2253	92	4.1	2373	104	4.4
Blinded placebo-controlled	312	33	10.6	326	34	10.4
Open add-on studies	1480	38	2.6	1575	54	3.4
Blinded active controlled	50	11	22.0	52	6	8.7
Open active controlled	411	10	2.4	420	10	2.4
Observational study	–	–	–	3873	0	0

Due to the frequent infectious origin of acute diarrhea in children, we specifically looked at efficacy of racecadotril in children with identified rotavirus infection; specific data related to other infectious causes of acute diarrhea were not identified. All data extractions from the manuscripts done by one of the authors were cross-checked by members of the Dept. of Pharmacology of the Johannes Gutenberg University (Mainz, Germany) as part of medical writing support.

### Statistical analysis and meta-analysis

**Fig. 2:**
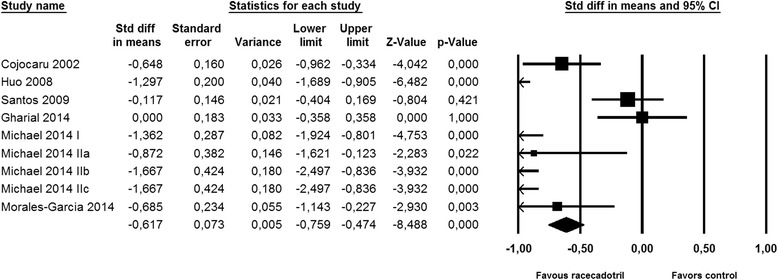
Effect of racecadotril in placebo-controlled and open-label add-on studies on number of stools on second day of treatment. Individual studies are depicted by a filled square, the overall estimate from the meta-analysis (fixed model) by a filled diamond in the bottom row. See also Fig. 7

**Fig. 3:**
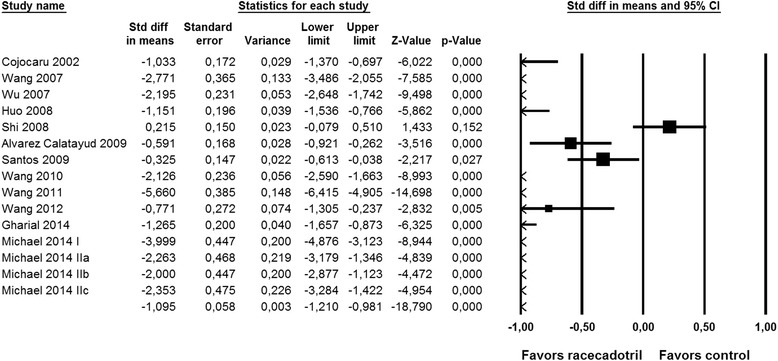
Effect of racecadotril in placebo-controlled and open-label add-on studies on time to cure. Individual studies are depicted by a filled square, the overall estimate from the meta-analysis (fixed model) by a filled diamond in the bottom row. See also Fig. 7

We have performed post-hoc statistical testing for the five efficacy parameters used at least 10 times and shown in Figs. 2 and 3 by performing paired, two-tailed t-tests using the Graphpad Prism software (version 7.0, Graphpad, La Jolla, CA, USA). Due to the post-hoc nature of the statistical tests, it should be noted that the resulting *P*-values are descriptive only and should not be interpreted as hypothesis testing. Therefore, we did not set a significance threshold but rather report exact P-values with three significant decimals. Descriptive P-values were not calculated for parameters used in less than 10 studies. Meta-analysis was performed for those five efficacy parameters using Comprehensive Meta-Analysis software (version 3.3.070, Biostat Inc., Englewood, NJ, USA) applying the fixed model procedure.

### General findings

Most of the 58 retrieved trials were reported from China (*n* = 44); others came from Egypt (*n* = 4), France (*n* = 3), Spain (*n* = 2) and Ecuador, Guatemala, India, Kenya and Peru (*n* = 1 each). Most studies were performed in hospital-based settings, others in office-based settings or combinations thereof (27, 8 and 6 studies, respectively), whereas 17 reports did not mention the study setting. While it can be assumed that office-based studies only recruited outpatients, some of the hospital-based studies apparently also included mainly outpatients [33–36]. Studies included sample sizes ranging from 15 to 165 patients per study arm, with 40–60 children per arm in most trials. Only few studies reported power calculations or other sample size justifications [33–35, 37–39]. Moreover, the specific randomization approach has been reported only rarely [34, 35, 39]. Studies covered a wide range of ages, starting as low as 1 month in some cases and ending as high as 10 years in one case. While duration of diarrhea prior to inclusion into the study varied, it was limited to not more than 3 days in most cases [24] and always comparable between the racecadotril and the comparator arm.

Studies assessed the effects of racecadotril as compared to placebo (*n* = 6), as add-on to various background treatments (*n* = 41) or relative to an active comparator (*n* = 15). Note that four studies compared racecadotril to more than one other treatment (Table 1). In line with national and international guidelines for the treatment of acute diarrhea in children [1, 2, 25], background treatment included ORT and/or intravenous rehydration treatment (IRT) in all cases with one exception [37]. Apparently related to local treatment standards, background treatment varied and additionally included the adsorptive agent smectite (*n* = 23), various probiotics (*n* = 22), anti-virals (*n* = 11), antibiotics (*n* = 3) and/or unspecified anti-infective agents (*n* = 3); one study each included zinc or non-specified anti-inflammatory background treatment, and one did not specify background treatment. Some studies included more than one of the above background treatments (Table 1). Active comparator treatments included the opioid receptor agonist loperamide (*n* = 1), smectite (*n* = 5), probiotics (*n* = 4), a combination of smectite and probiotics (*n* = 3), and a kaolin/pectin and lactose-free diet (*n* = 1 each). Of note, only six of all 58 studies were reported to be double-blind (5 vs. placebo, 1 vs. loperamide) and one was single-blinded, whereas all other randomized studies had an open-label design (Table 1).

The retrieved studies have reported a total of 45 different efficacy parameters (Table 2), most often duration of diarrhea/time to cure (*n* = 22), global status on day 3 as markedly effective/effective/ineffective (*n* = 30) or cure/improved/no change (*n* = 11), day 3 cure rate (*n* = 41) or as number of stools after 48 h (*n* = 12); for definition of global status see section 6.1. Cross-study comparisons for efficacy parameters reported in at least 10 studies are shown in Figs. 2 and 3. Many efficacy parameters were reported in only one (*n* = 16) or two reports (*n* = 9), making a cross-study comparison difficult for these parameters. Only 12 studies reported a defined primary efficacy parameter [33–41]. Sixteen studies did not report tolerability data, and two reported only qualitative tolerability data without providing specific incidences stratified by treatment.

## Results

### Blinded placebo-controlled studies

We have identified six randomized studies comparing racecadotril to placebo (5 double-blind, 1 single-blind; Table 1). They included a total of 312 patients in the placebo and 326 patients in the racecadotril arms, all with ORT and/or IRT background treatment. Four of them were of high quality including definition of a primary efficacy parameter [35, 38–40], sample size determination based on power analysis [35, 38, 39] and reporting of randomization procedure [35, 39], whereas one reported in abstract form only provided limited information [42]. The pre-specified primary endpoint of 48 h stool output was significantly reduced by 53% (331 ± 39 vs. 157 ± 27 g/kg with placebo and racecadotril, respectively) and by 60% (approximately 15 vs. 9 g/h with placebo and racecadotril, respectively), respectively, in the two studies reporting it [38, 40]. Two studies had a different pre-specified primary endpoint, number of stools on the second day of treatment; while racecadotril was superior to placebo in this regard in one study (4.1 ± 2.7 vs. 2.7 ± 1.5 stools) [35], it was not in another (5 vs. 5 stools) [39]. The latter differed from the five other placebo-controlled as well as most of the open-label studies in three ways: Firstly, this has apparently been the only racecadotril study including zinc supplementation as background treatment in both groups; given the limited effect of zinc according to a recent meta-analysis [43], this is not a likely factor to explain a difference. Second, the mean severity of the condition was higher than in most studies (mostly moderate to severe dehydration). Third and perhaps most importantly, treatment started later than in most studies [24], i.e. about half of all patients were included after 5 or more days of diarrhea.

Among reported other efficacy endpoints, a within-study statistically significant benefit of racecadotril as compared to placebo was reported for total stool output until cure, duration of diarrhea, time-to-cure in Kaplan-Meier analysis and volume of required ORT on day 2 of treatment [40], total stool output on first day of treatment and time-to-cure in Kaplan-Meier analysis [38], duration of diarrhea and total volume of required ORT [42], numbers of unformed stools, volume of required ORT and IRT, time to correct dehydration, global efficacy and overall 72 h cure rate [44], and number of stools on third treatment day and cure rate at 72 h [35]. On the other hand, the study not reaching its primary endpoint also found a lack of statistically significant differences between treatments for the secondary efficacy parameters, duration of hospitalization and time to cure [39].

In aggregate, these data demonstrate a superior efficacy of racecadotril as compared to placebo across a range of efficacy parameters in randomized, double-blind studies. Meta-analyses of two studies with high quality [38, 40] has previously confirmed the efficacy of racecadotril as compared to placebo [19, 21].

### Open-label add-on studies

Several open-label studies have explored racecadotril as an add-on to a background treatment. This was dominated by those from China, accounting for 34 of 41 studies. Background treatment consisted of fluid replacement only (ORT and/or IRT) in seven studies that included a total of 348 patients in the control and 350 in the racecadotril arms. Four of these had a specified primary endpoint; these were the need for a second emergency room visit after start of treatment [36], number of stools on second day of treatment [33, 34] and volume of stool output on second day of treatment [41]. Except for one study [34], the addition of racecadotril significantly improved the primary endpoint in all studies. The negative study had a different design as compared to the others as it included children who already had diarrhea for at least 7 days and required hospitalization. Significantly improved secondary endpoints included global efficacy after 3 days [45] and 5 days of treatment [46], number of stools on first [33, 41] and second day of treatment [36, 41], volume of stool output on first day of treatment [41], total volume of stool output until cure [41], time to cure [33, 36, 41, 45], cure rate after 3 days [45] or 5 days of treatment [46], cure rate after 3 days in subgroup with positive stool culture [34], total duration of diarrhea since onset of symptoms [45], duration of treatment [33], need for IRT [36], nursery/school attendance [33], number of patients (in %) with watery stools after 2 days [33], resolution of vomiting after 2 days [33], number of secondary doctor/emergency room visits [33], number of hospitalized patients after 24 and 48 h [33], and global satisfaction of physician and parents [33].

Five studies explored effects of racecadotril as an add-on to background treatment with fluid replacement plus the adsorptive agent smectite (Table 1). All of them describe % of patients reporting treatment to be markedly effective/effective/ineffective after 3 days and three studies on cure rate at that time point [30, 47–50]. Addition of racecadotril consistently improved these efficacy parameters in each of the five studies (*n* = 351 and 376 for background treatment and background with the addition of racecadotril, respectively). In one of the study, adding racecadotril to the fluid replacement plus smectite background also improved cure rate after 72 h, time to cure and duration of hospitalization [30].

Fifteen studies have explored the addition of racecadotril to a background treatment of fluid replacement, smectite and a probiotic (Table 1); while different probiotics have been used, they are discussed here together. They included a total of 846 and 734 patients receiving background treatment with and without additional racecadotril, respectively. Except for one study with efficacy assessment after 5 days of treatment [29], these studies consistently report a higher cure rate upon addition of racecadotril, typically increasing it from 60 to 80% to over 90% [51–64]. Racecadotril also consistently reduced time to cure [54, 64], duration of hospitalization [57, 58] and global efficacy assessment [51, 52, 54–64] in studies reporting these endpoints. Other endpoints improved by the addition of racecadotril included total disease duration [56], quantity of ORT requirement [53], duration of treatment [56] and time to cure fever [64].

Nine studies have explored the effect of racecadotril as an add-on to background treatment including an anti-viral agent (*n* = 455 and 492 for background treatment and racecadotril addition, respectively); this was specified to be ribaverine in one report [65], but in most cases, the specific anti-viral agent has not been reported. Other than the anti-viral agent, background treatments always included fluid replacement and sometimes smectite, a probiotic, an unspecified anti-infective agent or a combination thereof (Table 1). These studies consistently reported a superiority of racecadotril addition for global efficacy estimates [31, 65–72] and cure rate [31, 65–72] after 3 days of treatment. Additional endpoints with superiority of racecadotril included time to cure [65, 67, 71], total duration of disease [65, 67, 70, 71], duration of treatment [68, 70], duration of hospitalization [69] and blood levels of inflammatory markers such as interleukins 1, 8 and 12 [72].

Two small studies have included the antibiotics nitazoxanide or metronidazole as part of background treatment [41]. Both had a defined primary endpoint of time to cure, for which addition of racecadotril was statistically significantly superior (4.5 vs. 3.9 and 3.7 vs. 2.9 days, respectively; reported *P* < 0.01 for both studies). Racecadotril addition also was numerically superior for number of bowel movements after 24 and 48 h and cure rate after 7 days in both studies, but that did not reach statistical significance in all cases. One study applied racecadotril as addition to a background treatment of ORT and an unspecified anti-infective and reported superiority of racecadotril for global efficacy estimate and cure rate after 72 h [32]. A similar study additionally included a probiotic as part of background and also reported superiority of racecadotril addition for these two endpoints [73]. Finally, a study with poorly defined background treatment (described as “including such treatments as control of infections, maintenance of the electrolyte acid-base balance, microecological therapy and oral administration of intestinal mucosa protective agents”) also reported superiority of racecadotril addition after 72 h for global efficacy, cure rate and time to cure [74]. Taken together, a large number of studies consistently reported a beneficial effect of the addition of racecadotril to a wide range of standard treatments.

### Meta-analysis of placebo-controlled and add-on studies

To quantitatively analyze the effect of racecadotril in the placebo-controlled and add-on studies, we have performed meta-analysis for the most frequently used efficacy parameters (Table 2). This included only the placebo-controlled and add-on studies, which test the efficacy of racecadotril per se. In contrast, blinded or open-label actively controlled studies were not included in this comparison because differences between the variety of active controls would have introduced considerable heterogeneity.

Nine studies reported on number of stools on the second day after start of racecadotril administration. Although two studies with relatively large patient numbers [34, 39] did not reach intra-study statistical significance (both starting treatment considerably later after onset of symptoms), meta-analysis demonstrated a clear benefit of racecadotril on number of stools (Fig. 2). Fifteen studies reported on time to cure, among which only one did not show a statistically significant intra-study benefit [30]. Accordingly, meta-analysis demonstrated a major benefit of racecadotril on time to cure (Fig. 3).

**Fig. 4:**
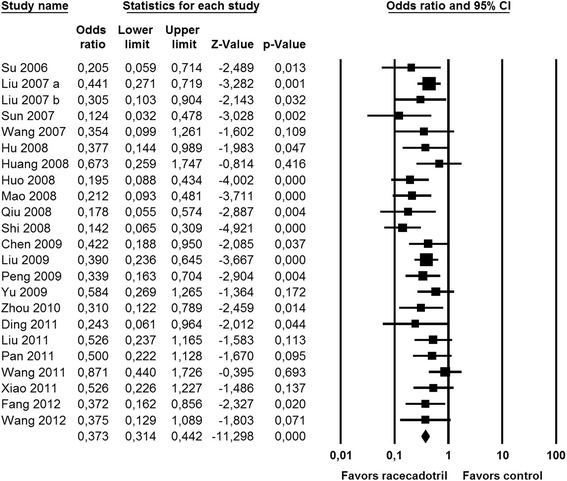
Effect of racecadotril in placebo-controlled and open-label add-on studies on global efficacy categorized as markedly effective, effective or ineffective. Odds ratios have been calculated for the outcome “markedly effective”. Individual studies are depicted by a filled square, the overall estimate from the meta-analysis (fixed model) by a filled diamond in the bottom row. See also Fig. 8

**Fig. 5:**
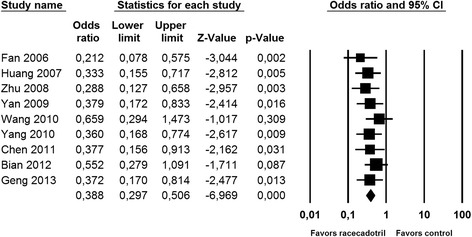
Effect of racecadotril in placebo-controlled and open-label add-on studies on global efficacy categorized as cured, improved or not-improved. Odds ratios have been calculated for the outcome “cured”. Individual studies are depicted by a filled square, the overall estimate from the meta-analysis (fixed model) by a filled diamond in the bottom row. See also Fig. 8

Many studies reported on global efficacy, categorized as markedly effective, effective or ineffective and assessed on day 3, an outcome definition defined and endorsed by the National Diarrhea Prevention and Treatment Commission in China [75]. This efficacy parameter was reported in 23 studies, including eight where numerical superiority did not translate to intra-study statistical significance. However, meta-analysis clearly demonstrated the benefit of racecadotril for this endpoint (Fig. 4). Nine studies have also used a global efficacy estimate but categorized it as cured, improved and not improved. While numerical improvement did not translate into intra-study statistical significance in two studies [65, 72], meta-analysis demonstrated a clear benefit of racecadotril for this global efficacy classification as well (Fig. 5).

**Fig. 6:**
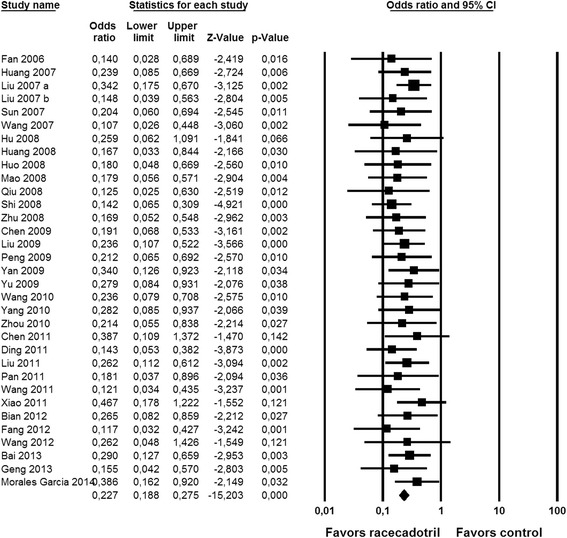
Effect of racecadotril in placebo-controlled and open-label add-on studies on % of patients cured after 72 h day of treatment. Individual studies are depicted by a filled square, the overall estimate from the meta-analysis (fixed model) by a filled diamond in the bottom row. See also Fig. 8

Finally, 33 trials used cure rate on day 3 of racecadotril treatment as an outcome parameter. While all of them reported numerical superiority, this did not reach intra-study statistical significance in four of them. Accordingly, meta-analysis demonstrated a major benefit of racecadotril for this global efficacy scale as well (Fig. 6).

### Blinded actively controlled studies

We have identified only one double-blind, actively controlled multi-center study, which compared racecadotril with loperamide treatment [37]. The study was of high quality due to blinding, definition of a primary endpoint (number of diarrheic stools until recovery), and sample size calculation. Due to the regulatory restriction not to use loperamide in children younger than 2 years [3], this study recruited a considerably older population than all other studies (Table 1). Also because of regulatory restrictions in the use of loperamide, the study excluded patients having received any antibiotic in the past 30 days or having a current need for antibiotic treatment. Each patient received the respective active treatment plus a placebo matching the other active treatment. The two treatment groups did not differ significantly for the primary endpoint of number of diarrheic stools until recovery (2.7 ± 0.4 stools for racecadotril vs. 2.1 ± 0.4 stools for loperamide) and for secondary endpoints duration of diarrhea (10.7 ± 1.7 vs. 8.8 ± 2.3 h) and recurrence rate (22% vs. 19%). Therefore, the two treatments were concluded to have similar efficacy. However, to achieve this comparable efficacy, almost three times as many patients in the loperamide group required concomitant other medications (8 with anti-emetics, 3 with analgesics, 2 with ORT, and 1 with laxative) as in the racecadotril group (5 with anti-emetics).

### Open-label actively controlled studies

Five studies compared the efficacy of racecadotril to that of smectite (total of 399 vs. 395 patients), three of which included a probiotic as part of shared background treatment (Table 1). Two of these studies reported a comparable efficacy of racecadotril and smectite, for instance for global efficacy and cure rate after 72 h [30, 76] and in one of these studies also for time to cure and duration of hospitalization [30]. Of note, one of these studies also had included an arm with combination of smectite and racecadotril and found this to be more effective than either monotherapy [30]. Three other studies, however, reported a superiority of racecadotril over smectite for global efficacy [77, 78], cure rate after 72 h [77, 78], number of stools on the first, second and third day of treatment [79], time to cure [79], duration of treatment [79] and duration of hospitalization [77].

Four studies have compared the efficacy of racecadotril (*n* = 182 patients) with that of a probiotic (*n* = 180 patients), in some cases including an anti-viral or an unspecified anti-infective drug as part of background treatment (Table 1). Two of them reported comparable efficacy of the probiotic and racecadotril for global efficacy estimates and day three cure rate [31, 32], whereas two others reported significant superiority of racecadotril for these parameters [80, 81]. Interestingly, both studies showing comparative efficacy of the tested probiotic and racecadotril found that combination treatment yielded a significantly greater efficacy than either monotherapy. One of the two studies reporting a superior efficacy of racecadotril also reported superiority for the additional endpoints time to cure, duration of dehydration, duration of fever and total duration of disease from start of symptoms [81].

Three studies, using only fluid replacement as background treatment (and an unspecified anti-viral in one study [82]) have compared racecadotril to a combination of smectite and probiotics (total of 152 and 145 patients, respectively). While the two studies evaluating efficacy after 3 days of treatment reported superiority of racecadotril for cure rate and global efficacy [82, 83], the study with efficacy assessment after 5 days of treatment did not find significant differences for these parameters [29]; it also found no significant difference for combination treatment as compared to the two other study arms.

Two studies have evaluated racecadotril vs. active comparators not otherwise tested. One explored the effects of racecadotril as compared to those of treatment with a kaolin/pectin combination [84]. It reported superior efficacy of racecadotril for number of stools after 24 h (5.5 vs. 8.9 stools) and 48 h of treatment (3.0 vs. 6.3 stools), total intake of rehydration (1140 vs. 1870 mL), duration of diarrhea (30 vs. 42 h) and total number of stools until recovery (8.9 vs. 19.0 stools). The other, performed in a rotavirus-positive population and using a background treatment of fluid replacement, smectite and a probiotic, compared racecadotril to a lactose-free diet and a combination of both treatments [85]. For each of the reported endpoints, racecadotril was more effective than lactose-free diet but combination was most effective; these included global efficacy rated as markedly effective/effective/ineffective (23.7/44.7/31.6% for racecadotril vs. 14.7/26.5/58.8% for lactose-free diet vs. 40.9/50/9.1% for combination treatment) and cure rate (68.4% vs. 41.2% vs. 90.9%), duration of IRT requirement (3.5 vs. 4.2 vs. 2.1 h), and treatment duration (4.2 days vs. 5.5 days vs. 3.2 days).

### Tolerability of Racecadotril

The six placebo-controlled studies with a total of 326 and 312 patients in the racecadotril and placebo groups, respectively, reported a total 34 adverse events (AEs) in the racecadotril and 33 in the placebo arms (10.4% vs. 10.6%), respectively, demonstrating that overall AE incidence with racecadotril was similar to that with placebo [35, 38–40, 42, 44]. In one of the studies, three children had to be withdrawn due to convulsions, all of which occurred in the racecadotril group [39]; however, this finding is difficult to interpret as convulsions were not reported with racecadotril treatment in any of the other placebo-controlled, add-on or active comparator studies (Table 1) or in a large open-label study [27], covering a total of more than 7000 children exposed to racecadotril. The three cases of convulsions in the racecadotril group of one study are also difficult to interpret because three cases in 7000 patients with a short treatment period would indicate a higher incidence than in the general population up to age 17 years [86]; on the other hand, acute diarrhea is often accompanied with fever and, particularly in a 5–60 months old population as in that study, fever per se is a leading cause of convulsions.

The studies exploring the addition of racecadotril to fluid replacement reported a total number of 19 AEs in the control arms (5.5%) and 23 AEs in the racecadotril arms (6.6%) [33, 34, 36, 41, 45]. While most AEs were mild, one study reported two serious AEs in the control (one hospitalization each due to vomiting and dehydration) and one in the racecadotril arm (one transaminase elevation attributed to suspected virus infection (ALT 957 IU/L; AST 1357 IU/L)), respectively [34]. One study reported comparable occurrence of rebound constipation in both arms (18.6% vs. 20.3% with racecadotril and background treatment, respectively) [33]. In the four studies with the addition of racecadotril to a background treatment including smectite [47–50], the total incidence of AEs across studies was 7 in each group (2.7% vs. 2.4% with placebo and racecadotril, respectively), driven by a comparable incidence of rebound constipation (5 vs. 4 children). The studies with the addition of racecadotril to a background treatment of fluid replacement, smectite and a probiotic [29, 52, 54–63] reported a total AE incidence in the background and racecadotril groups of 1.3 and 2.8%, respectively. The studies with background treatment including an anti-viral agent [65–67, 69–71] had a total AE incidence with background treatment and racecadotril of 1.7 and 2.1%, respectively. Two small studies with background treatment including an antibiotic found an AE incidence of 0% in both study arms [41]. The AE incidence across all add-on studies was 3.6% with racecadotril and 2.4% with the various background treatments (Table 3).

The only blinded study comparing racecadotril to another active treatment reported an AE incidence of 22% with loperamide and 11.5% with racecadotril [37]. One serious AE occurred in the loperamide group, emergency hospitalization due to fever development. Vomiting was the most frequent AE in both groups (*n* = 5 and 4, respectively). Constipation was not counted as AE by the investigators but was significantly more frequent with loperamide than with racecadotril (58% vs. 36.5%). When occurring, the duration of constipation was similar in both groups (1.8 vs. 1.6 days). Abdominal circumference was comparable between groups in this pediatric study, whereas studies in adults had consistently found a quicker resolution of diarrhea-associated meteorism with racecadotril than with loperamide [26].

The studies comparing racecadotril to smectite reported an AE incidence of 1.9 and 0.8%, respectively [76, 77, 79]. None of the trials comparing racecadotril to a probiotic reported on AE incidence [31, 32, 80, 81]. In studies comparing racecadotril to a smectite plus probiotic combination [29, 82, 83], AE incidence was 3.3% as compared to 5.5%. The accumulated AE incidence in all non-blinded studies comparing racecadotril to an active comparator was 2.4% in both groups (Table 3).

## Discussion

### Critique of methods and limitations of analysis

Our analysis is based on a systematic search for randomized studies testing the efficacy and tolerability of racecadotril in the treatment of acute diarrhea in children (Fig. 1). This yielded a total of 60 studies, but we have been unable to retrieve data for two of them despite intensive efforts. While omission of these two trials is regrettable, the remaining 58 trials compare represent a much larger evidence base than any of the previous reviews in the field that have covered no more than 19 [24] and in all other cases less than 10 studies. A driver of the much larger number of retrieved trials was the inclusion of studies irrespective of country of origin and language of publication. Specifically, previous reviews in the field have not included most studies from Asia and many from Africa and Latin America. This is unjustified as acute diarrhea represents a major share of the global disease burden on these continents. Moreover, many countries in Asia, Africa and Latin America have healthcare systems with limited resources. This makes it imperative for an impact on the global health that a treatment shows efficacy under those challenging conditions. Thus, we consider it a major strength of our analysis to provide the first truly comprehensive and global review of the topic using Preferred Reporting Items for Systematic Reviews and Meta-Analyses (PRISMA; www.prisma-statement.org).

Some studies had high technical quality, particularly those comparing racecadotril to placebo or to loperamide. This included blinding, pre-specified primary endpoints, sample size determination based on power calculation and description of randomization approach. However, blinding had only been applied to 7 of 58 randomized racecadotril studies. Specifically, only 1 of 44 studies from China was blinded. As China accounted for 76% of all randomized studies, exclusion of the open-label studies would have ignored a major part of the overall evidence. Another sign of moderate study quality is the fact that 13 of the 58 retrieved studies did not report on tolerability of the treatment under consideration, and two reported only qualitative data without specific allocation to treatment groups. The lower evidence quality associated with these factors needs to be taken into consideration in the interpretation of the resulting data.

Another limitation of the existing literature is that most racecadotril studies, similar to those for other types of acute diarrhea medications [87], have been quite small (Table 1). Large sample sizes may not be required in fields where the difference in efficacy between treatments is large. However, studies with mostly small sample sizes increase the probability of a reporting bias. It should be noted that according to our meta-analysis for the five most frequently applied efficacy parameters, the percentage of studies not demonstrating statistically significance for an endpoint ranged from 7 to 35%, indicating that negative data were reported. In most of these “negative” studies, racecadotril was numerically superior to control but this could not be verified statistically with small sample sizes. Accordingly, meta-analysis for all five frequently used endpoints clearly demonstrated considerably greater efficacy of racecadotril as compared to placebo or when given as add-on treatment. Consistency across so many studies is difficult to explain by reporting bias. The inclusion of a much larger number of studies than previous reviews representing data from about 2500 patients each in the racecadotril and comparator arms provides solid clinical evidence despite limited sample size in many individual studies.

It is noteworthy that the randomized studies summarized here have included vastly different background treatments. On the one hand, this can be seen as a strength of the available evidence, i.e. that the effectiveness of racecadotril is very robust because it has been observed against a large variety of background treatments. On the other hand, this heterogeneity may make network meta-analysis approaches more suitable than the classic meta-analysis calculations reported here. While beyond the scope of the present project, such network meta-analysis has been applied to a limited number of racecadotril studies in the past [43], and application of such techniques to the broader range of studies reported here will be useful to the field.

Adherence to global recommendations for non-pharmaceutical treatment of acute diarrhea in children is a sign of good study design, specifically the recommendation for fluid and electrolyte replacement as the foundation of any other intervention [1, 2, 25, 87–89]. With one exception [37], all studies in children with acute diarrhea have explored efficacy and tolerability of racecadotril in addition to oral and/or intravenous rehydration treatment. In contrast, placebo-controlled studies in adults with acute diarrhea with one exception [90] have not used systematic rehydration treatment in most other studies [17, 91–93].

About 75% of all studies have reported on global efficacy of racecadotril, mostly assessed after three but in few cases also after 5 days of treatment (Table 2). These global efficacy estimates are based on categorical classification of treatment outcome as markedly effective/effective/ineffective or as cured/improved/not improved (Fig. 3). Almost all studies applying such global efficacy estimates came from China. The National Diarrhea Prevention and Treatment Commission in China endorses such global efficacy assessment and has issued a formal definition [75]. According to this definition, ‘markedly effective’ means that diarrhea frequency was reduced to < 2 times per day within 24–48 h of medication, the water content had clearly decreased, the stool routine microscopy test was positive, the stool had a fully formed or soft appearance, and the clinical symptoms had completely disappeared; ‘effective’ means that diarrhea was reduced to < 4 times per day within 48–72 h of medication, the water content had clearly decreased, the stool microscopy test was negative, and the clinical symptoms had essentially disappeared; ‘ineffective’ means that there was no alleviation in diarrhea within 72 h, it even worsened in some cases, and there was no change in general symptoms. Some reports explicitly indicate to have applied this definition [31, 67, 74, 85]. However, many others do not provide a definition of global efficacy or state to have applied alternative definitions such as ‘markedly effective’ meaning that frequency of defecation had declined to not more than two stools/day [76] or as negative stool culture at 72 h [46]. The strength of providing a global efficacy estimate is that it provides an intuitive impression of efficacy; the weakness is that it can be somewhat subjective. The latter can particularly be a problem in open-label studies.

**Fig. 7:**
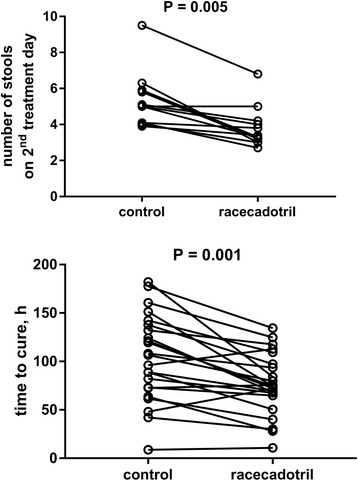
Effect of racecadotril as compared to comparator treatment on number of stools on 2nd day of treatment (upper panel) and time from start of treatment to end of diarrhea (‘time to cure’, lower panel). Each pair of data points represents one study. Note that comparator treatments included placebo (*n* = 2 and 3 studies, respectively), various background treatments (*n* = 8 and 16 studies, respectively) and active comparator treatments (*n* = 3 and 5 studies, respectively); for details see section 4. Descriptive *P*-values are based on paired, two-tailed t-tests

**Fig. 8:**
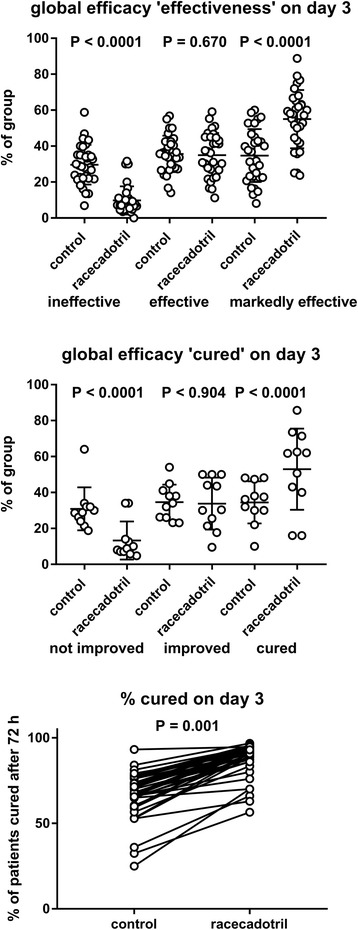
Effect of racecadotril on global parameters of efficacy assessed 72 h after start of treatment. Shown are degree of improvement classified as ‘ineffective’, ‘effective’ and ‘markedly effective’ (upper panel), as ‘not improved’, ‘improved’ and ‘cured’ (middle panel) and as % of patient considered cured at end of third treatment day (lower panel Each data point represents one study; for lower panel data points within a study are connected by a line. Note that comparator treatments included placebo (*n* = 1, 2 and 2 studies, respectively), various background treatments (*n* = 22, 9 and 30 studies, respectively) and active comparator treatments (*n* = 9, 2 and 10 studies, respectively); for details see section 4. Descriptive *P*-values are based on paired, two-tailed t-tests comparing % values within a category between the two treatments

Investigators have applied a plethora of other efficacy parameters (Table 2). Many of these efficacy parameters are informative, but if they have been used in only one or two studies, a robust assessment of the efficacy of racecadotril for that parameter is difficult. Therefore, our analysis has focused on the parameters applied in at least 10 studies, and the corresponding data are depicted in Figs. 7 and 8 (see section 6.2).

The pharmaceutical industry has been criticized in the past for lack of transparency on clinical trials and non-reporting of ‘negative’ studies [94]. The introduction of clinical trial registries such as clinicaltrials.gov has improved this situation. In this regard, it is noteworthy, that only some of the blinded and none of the randomized open-label studies apparently had been industry-sponsored. Therefore, the existing literature can be expected to exhibit little bias against ‘negative’ findings based on commercial interest; bias against ‘negative’ studies due to other factors cannot be excluded but is not likely to play a major role given the large number of studies and the wide range of efficacy parameters they have used.

Given the strong evidence for efficacy of racecadotril, we have taken a closer look at the “negative” studies. In most cases, numerical differences of a clinically relevant magnitude did not reach statistical significance with small sample sizes whereas improvements for other efficacy parameters were seen. Only two studies did not observe efficacy of racecadotril. They had reasonable sample sizes but differed from all others by including patients 5–7 days after onset of symptoms [34, 39]. This raises the possibility that treatment with racecadotril should start early of onset of symptoms for optimal efficacy.

### Efficacy considerations, including patient subgroups

Among the 45 efficacy parameters being used in the 58 studies, five were used in at least 10 studies each. A meta-analysis of the placebo-controlled or add-on studies using these parameters has been described in section 4.3 and showed benefit of racecadotril treatment for each of them. To further explore the efficacy of racecadotril, we have also performed descriptive statistical analysis of studies reporting on these parameters including also the actively controlled trials.

As acute diarrhea is a self-limiting condition, duration of disease/time to cure from start of treatment may be the most relevant efficacy parameter from a patient perspective. Data on time to cure have been reported from 24 comparisons in 23 studies [30, 33, 34, 36, 37, 39–42, 45, 54, 64, 65, 67, 71, 74, 79, 81, 84], including three reported in the same paper in which it has been the pre-specified primary endpoint [41] and two comparisons in a 3-armed study [30]. Nineteen of these 24 comparisons reported a shorter duration of disease with racecadotril than with the comparator treatment, including the three studies where time to cure had been the pre-specified primary endpoint (Fig. 7). In the average of all studies, racecadotril treatment reduced duration of disease from 106.2 h to 78.2 h (mean reduction 28.0 h (confidence interval 16.4–39.6 h; *P* < 0.0001 in a two-tailed, paired t-test). Seven and six studies (not necessarily the same showing time to cure data) reported on two related parameters, i.e. total duration of diarrhea from start of symptoms [45, 56, 65, 67, 70, 71, 81] and/or on duration of treatment [33, 56, 68, 70, 79, 85], respectively; they consistently reported superiority of racecadotril over the comparator treatment. Interestingly, a non-interventional study based on 3873 children aged 3 months to 12 years seen by 97 pediatricians in Venezuela found a mean time to relief of 18.5 h (confidence interval 17.9–19.0 h) [27]. This is considerably faster than the 78.2 h observed in the randomized studies; however, in this observational study time from start of symptoms to start of treatment was only 7.9 h (confidence interval 7.3–8.6 h), i.e. much shorter than in most randomized studies. In a multiple regression analysis within the observational study, only diarrhea severity prior to start of treatment significantly affected time to relief.

A related efficacy parameter with high patient-relevance is the percentage of children cured after 3 days of treatment; this has been applied in 42 comparisons from 41 studies [30–32, 35, 44, 45, 48–74, 76–78, 80–83, 85] including one 3-armed study [30]. Except for one study demonstrating a minor numerical superiority of racecadotril without reaching statistical significance [76], all studies consistently reported a greater percentage of children considered cured after 3 days of treatment with racecadotril than with comparator treatment (88.3%vs. 67.4%; mean difference 20.8%; confidence interval 18.1–23.5%; *P* < 0.0001 in a two-tailed, paired t-test; Fig. 8). Percentage of recovered children after 5 days of treatment exhibited a less clear picture with three studies showing an advantage of racecadotril [40, 46], one a numerical but non-significant advantage for racecadotril [29] and one a numerical but non-significant advantage for the comparator treatment [38]. Cure rates assessed after 7 days of treatment exhibited small but consistent differences in favor of racecadotril vs. comparator treatments in four studies [34, 41]. To shed more light on the time course of resolution of diarrhea upon treatment with racecadotril as compared to placebo, two double-blind studies documented Kaplan-Meier analysis for 4–5 days after start of treatment [38, 40]. While only one of them detected greater recovery rate with racecadotril as compared to placebo at study end, both demonstrated a significantly faster resolution with racecadotril as compared to placebo treatment.

The second most frequently reported efficacy parameter, used in 32 comparisons in 30 studies, was global efficacy after 3 days of treatment rated as very effective/effective/not effective [30, 31, 44, 45, 47–55, 57, 59, 61–64, 66, 67, 71, 74, 76–78, 80, 82, 83, 85] including two 3-armed studies [30, 31]. Consistently across all 32 comparisons, racecadotril relative to comparator had fewer children rated as “not effective” (mean difference 19.9%; confidence interval 16.6–23.1%; *P* < 0.0001 in a two-tailed, paired t-test) and more rated as “very effective” (mean difference 20.3%; confidence interval 16.4–24.2%; *P* < 0.0001; Fig. 8). The global efficacy after 3 days of treatment rated as cured/improved/not improved was used in 11 studies [32, 56, 58, 60, 65, 68–70, 72, 73, 81]. With the exception of the probiotic vs. racecadotril arm of one study [32], these studies consistently reported superiority of racecadotril relative to the comparator treatment for percentage of patients ‘not improved’ (mean difference 17.6%; confidence interval 10.7–24.6%; *P* = 0.0002) and more patients ‘cured’ (mean difference 18.5%; confidence interval 10.1–26.8%; *P* = 0.0006; Fig. 8).

Frequently used objective parameters of treatment efficacy were number of stools at various time points after start of treatment. Twelve studies have reported on number of stools on day 2 of treatment [33–36, 39, 41, 54, 79, 84], including 4 where it was the pre-specified primary endpoint [33–35, 39]. Except for one neutral study [39], treatment with racecadotril yielded fewer stools on the second day of treatment than the comparator (3.8 vs. 5.3; mean difference 1.5; confidence interval 0.8–2.2; *P* = 0.0005; Fig. 7). Although less frequently reported, number of liquid/watery stools may be more relevant for patients and physicians than total number of stools, and some studies explicitly report on that. Two studies comparing racecadotril + ORT to ORT alone have explicitly reported on number of liquid/watery stools on the second day of treatment; one reported that racecadotril reduced the percentage of children with watery stools from 77.9 to 40.7% [33], whereas the other found a comparable percentage in both groups [34]. While many studies explicitly had included only children with watery diarrhea [29, 38–40, 42, 44, 51, 63, 66, 82, 83, 95], they unfortunately did not report specific outcomes for liquid stools. Taken together, these studies demonstrate a highly consistent advantage of racecadotril over various comparator treatments of acute diarrhea in children, irrespective of efficacy parameter being applied and in both blinded, placebo-controlled and open-label randomized studies.

Acute diarrhea in children most often is of infectious origin, and both viruses and bacteria may be the causative agent. While some studies have reported prevalence of certain bacteria at baseline [34, 40], most did not report on specific outcomes per bacteriological status. One trial found that racecadotril as compared to fluid replacement only reduced bowel movements after 48 h in children with bacteria-positive stools [34]. As rotavirus infection is a common cause of acute diarrhea in children and has a major socio-economic impact [2, 96], several studies specifically reported efficacy in rotavirus-positive populations [30, 31, 45, 52, 58, 59, 64, 65, 70, 71, 74, 81, 85]. These provided similarly consistent evidence for superiority of racecadotril over comparator treatment as the overall studies. Perhaps more interesting are intra-study comparisons of racecadotril efficacy in rotavirus-positive and -negative children. While one of them found duration of disease to be longer in rotavirus-positive and -negative boys being treated with placebo, this difference according to viral status was not present in the racecadotril-treated groups; it was shorter with racecadotril than with placebo in either group. Three other studies, applying different efficacy parameters, reported a similar efficacy of racecadotril in rotavirus-positive and -negative children [33, 38, 41]. Interestingly, one study found that negative stool cultures after treatment were found more frequently with racecadotril than with fluid replacement only (37.5% vs. 16.7%) [46].

Whether children with acute diarrhea are being treated as outpatients or being hospitalized depends on several factors, including severity of symptoms and the structure of the local healthcare system. In this regard, the 2008 joint guidelines of the European Society for Paediatric Gastroenterology, Hepatology and Nutrition and European Society for Paediatric Infectious Diseases had recommended the use of racecadotril but concluded that insufficient data were available to support the use of racecadotril in outpatient settings [97]. In the 2014 update of this guidelines, the limitation on limited outpatient data was no longer mentioned but the overall database was still considered limited [2]. While most studies reported whether the participating physician was hospital- or office-based (Table 1), it was not always clear whether hospital-based physicians treated diarrhea primarily in outpatients or after hospitalization. It can safely be assumed that all office-based studies were performed on outpatients [41, 48, 64, 76, 84, 85]. Taken together, these studies present evidence in favor of racecadotril efficacy in outpatients based on 254 and 246 being treated with racecadotril and comparator, respectively, in a total of 8 studies. If one additionally considers trials performed by hospital-based physicians but explicitly stating that they included only outpatients [33, 34] or reporting typical outpatient efficacy parameters such as second emergency room visit [35, 36], the total number of outpatients receiving racecadotril or comparator increases to at least 544 and 535, respectively. While we do not know which fraction of the patients in the trials involving hospital- and office-based physicians involved outpatients [29, 32, 49, 60, 71, 82], this may even increase number of outpatients receiving racecadotril or comparator to up to 927 and 905, respectively. In this subgroup of studies, efficacy parameters were similarly favorable relative to the comparator treatments as in the overall group of trials. Therefore, the current analysis firmly establishes the efficacy of racecadotril as compared to comparator treatments in outpatient settings.

While loperamide is a standard treatment for mitigating symptoms in adults with acute diarrhea [1], its use in children is excluded in those aged less than 24 months [3] and recent guidelines more generally recommend not to use loperamide in children [2]. Of note, most racecadotril studies in acute diarrhea have included children of less than 24 months (Table 1). Our search has identified only one study comparing racecadotril with loperamide in children with acute diarrhea [37]. That trial found loperamide and racecadotril to be equally effective for the primary endpoint, number of diarrheic stools until recovery, and for secondary endpoints, but significantly more children in the loperamide group required concomitant medications to reach this goal. While it may be argued that loperamide may have been under-dosed in that study (0.03 mg/kg as compared to regular dose of 0.04 mg/kg), the loperamide dose was sufficient to cause a considerable incidence of AEs. Moreover, the finding of comparable efficacy of loperamide and racecadotril is in line with many direct comparative trials in adults [98–103]. Thus, in contrast to loperamide, there is a solid database supporting the use of racecadotril in children of all ages including those aged less than 24 months. In an age group where both treatments can be used, they exhibit similarly efficacy, as they did in many studies in adults.

### Tolerability considerations

The most objective assessment of tolerability derives from blinded studies. In this regard, six blinded studies including 326 and 312 patients in their combined racecadotril and placebo arms have reported a cumulated AE incidence of 10.4 and 10.6%, respectively (Table 3). A double-blind trial comparing racecadotril to loperamide included on 52 and 50 patients per arm, respectively, but even with these limited sample sizes detected a statistically significant difference in AE incidence (11.5% vs. 22.0%) [37]. Open add-on studies with 1480 patients receiving background treatment only and 1575 children receiving additional racecadotril reported also a low AE incidence with racecadotril (3.4%; Table 3); this was lower than in the placebo-controlled trials but only slightly higher than with background treatment. A minor increase with active treatment in an open-label study may at least partly represent observer bias. The latter conclusion is supported by the observation that open actively controlled studies including 420 and 411 patients found an identical AE incidence with both active treatments (Table 3). Thus, across all studies, racecadotril and its various comparators had a comparable AE incidence (4.4% vs. 4.1%; Table 3). Given that this is based on more than 2000 participants per arm, these are robust numbers despite the fact that 22% of studies did not report on AE incidence. A formal meta-analysis based on four placebo-controlled or add-on studies [34, 36, 38, 40] also found that AE incidence occurred similarly in the absence and presence of racecadotril (hazard ratio 0.99, confidence interval 0.67–1.46) [21]. A second meta-analysis apparently being based on a larger number of studies but reported in abstract form only [104] reported the relative risk for experiencing an AE with racecadotril as compared to placebo to be 0.765 (confidence interval 0.611–0.962). The most recent meta-analysis, based on four studies in five distinct populations reported a risk ratio of 0.99 (confidence interval 0.73–1.34) [23].

Vomiting is a symptom frequently associated with acute diarrhea. While most investigators did not report specific incidence of vomiting, some counted vomiting as AE [29, 37, 38, 69] whereas others reported it but did not consider it as AE [35, 40]. The overall incidence of vomiting in the racecadotril arm ranged from 1.5% [69] to 51.5% [40], complicating cross-study comparisons between treatments. The five trials with specific vomiting data reported a combined incidence in 59 of 308 patients in the racecadotril and 53 of 331 patients in the comparator arms (19.2% vs. 16.0%). Of note, incidence of vomiting in both groups was driven by a single placebo-controlled study contributing 35 cases of vomiting to each arm [40]. Thus, it appears that vomiting occurs with comparable incidence with racecadotril and comparator treatments but more data will be required for a robust conclusion.

Rebound constipation can occur once acute diarrhea has resolved. In adults, the frequency of rebound constipation is markedly increased upon treatment with loperamide whereas racecadotril does not cause rebound constipation as compared to placebo [26]. Rebound constipation is not only unpleasant but can become medically relevant by retaining the viruses and bacteria having caused diarrhea in the gut [105]. In this regard, loperamide but not racecadotril has been shown to promote gut retention of infectious agents in animal models [18]. In our analysis of randomized trials evaluating racecadotril in the treatment of acute diarrhea in children, nine studies explicitly reported on constipation incidence [33, 37, 48, 54, 57, 60, 63, 67, 79]. In the combined racecadotril and comparator arms, the incidence was 39 of 551 patients and 53 of 536 patients (7.1% vs. 9.9%). When the blinded study comparing racecadotril with loperamide [37] was excluded from the analysis, constipation incidence dropped to 4.0% vs. 4.9% in the racecadotril and comparator arms, respectively. Taken together, these data clearly demonstrate that racecadotril does not cause rebound constipation.

## Conclusions

Acute diarrhea is a frequent condition in children, a leading cause of hospitalization and, particularly in countries with developing healthcare infrastructures, a relevant cause of mortality [106]. Recent guidelines issued by learned societies and other academic bodies recommend racecadotril as an option in the treatment of acute diarrhea in children [1, 2, 25]. Exceptions are guidelines from countries where racecadotril is not available, for instance those of NICE in the UK [89] and the Center of Disease Control in the US [87], and/or those issued more than a decade ago when the clinical evidence related to racecadotril was limited [87, 88]. While the efficacy and tolerability of racecadotril in the treatment of acute diarrhea in children has been reviewed repeatedly [19–23, 43, 107, 108], these earlier reviews covered less than a third of the available literature and often underrepresented evidence from developing countries. This is unfortunate given the specific societal impact of acute diarrhea in developing countries [106]. By not having a bias for country of origin and reporting language, we could evaluate 58 randomized studies exploring the efficacy and tolerability of racecadotril as compared to placebo or active treatments or given as add-on to various types of standard treatment. In line with previous reviews and meta-analyses [19–23, 43, 107, 108], our review demonstrates the efficacy and tolerability of racecadotril as compared to a wide range of other treatment options but bases these conclusions on a much larger body of evidence. This cumulative evidence reinforces the conclusion from guidelines based on a more limited analysis of the existing literature [1, 2, 25]. Thus, based on a large body of evidence regarding efficacy and tolerability, racecadotril is a valuable therapeutic option for the treatment of acute diarrhea in children.

## Abbreviations

AE: Adverse event
IRT: Intravenous rehydration treatment
ORT: Oral rehydration treatment
